# Pedicle screw placement accuracy of bone-mounted miniature robot system

**DOI:** 10.1097/MD.0000000000005835

**Published:** 2017-01-20

**Authors:** Tai-Hsin Tsai, Rong-Dar Tzou, Yu-Feng Su, Chieh-Hsin Wu, Cheng-Yu Tsai, Chih-Lung Lin

**Affiliations:** a Graduate Institute of Medicine, College of Medicine, Kaohsiung Medical University; b Department of Neurosurgery, Kaohsiung Medical University Hospital; c Graduate Institute of Clinical Medicine, College of Medicine, Kaohsiung Medical University, Kaohsiung, Taiwan.

**Keywords:** accuracy, bone-mounted miniature robot system, pedicle screw

## Abstract

This article describes factors affecting the accuracy of transpedicle screw placements performed with the Renaissance robot-guided system and reviews the relevant literature. Between January 2013 and January 2015, Renaissance robot-guided spinal surgery was performed in 125 patients at Kaohsiung Medical University Hospital in Kaohsiung, Taiwan. The surgeries included 662 transpedicle screw implants and 49 Kirschner wire (K-wire) reimplants performed by intraoperative repositioning. The lead author evaluated the accuracy of all K-wire insertions and classified their accuracy into 3 categories relative to the preoperative plan for transpedicle screw placement. For cases in which screws required repositioning after the registration step, factors affecting pedicle screw placement were determined according to the consensus of 3 experienced spinal surgeons. According to the scheme developed by Kuo *et al* (PLoS One 2016;11:e0153235), the K-wire placement accuracies before and after repositioning were respectively classified as follows: 76.1% and 77.6% in type I; 12.2% and 17.7% in type IIa; 4.3% and 4.5% in type IIb; 6.4% and 0% in type IIIa; and 1% and 1% in type IIIb. The percentage of screws requiring repositioning due to drilling error was 85.7% (42/49). Comparisons of preoperative and postoperative function showed significantly improved accuracy. This study showed that inaccurate pedicle screw placement mainly results from errors in preoperative planning, mounting, registration, drilling, and robot assembly. Pedicle screw placement using a bone-mounted miniature robot system requires meticulous preoperative planning to minimize these errors.

## 1 Introduction

Transpedicle screw placement, which was first described by Roy-Camille et al,^[1]^ is among the most effective schemes for stabilizing the spine. Nonguided methods of transpedicle screw implantation^[2]^ have a high risk of inaccurate screw positioning, which can cause severe neurovascular complications. Therefore, various guided methods of transpedicle screw implantation have been developed, including fluoroscope-guided placement,^[3]^ navigation-guided placement,^[4,5]^ and Renaissance robot-guided placement.^[6,7]^ The accuracy of the Renaissance robot-guided system is considered satisfactory^[8,9]^ but can still be improved by optimizing certain factors.

This article describes factors affecting the accuracy of transpedicle screw placements performed with the Renaissance robot-guided system and reviews the relevant literature.

## 2 Materials and methods

### 2.1 Inclusion and exclusion criteria

We retrospectively analyzed patients who had received robot-assisted transpedicular screw fixation to correct degenerative lumbar spondylosis or spondylolisthesis at Kaohsiung Medical University Hospital between January 2013 and January 2015. Indications for robot-assisted surgery were failure of conservative treatment, ongoing neurological deficit, intractable back pain, and progression of deformity. Inclusion criteria were diagnosis of degenerative lumbar disease, age >20 years at the time of diagnosis, a condition refractory to conservative treatment for at least 6 months, correction by robot-assisted pedicle screw placement, and follow-up >12 months. Patients were excluded if they had any history of spinal trauma, spinal infection, spinal malignancy, or adult degenerative scoliosis. Out of (number) patients who met the inclusion criteria, (number) were excluded. Ten patients were lost to follow up. Therefore, the final analysis included 125 patients.

### 2.2 Ethics statement

This clinical study was approved by the Institutional Review Board of Kaohsiung Medical University Hospital (KMUHIRB-E(I)-20150167). Written informed consent was obtained from all participants.

### 2.3 Clinical characteristics

**Figure 1:**
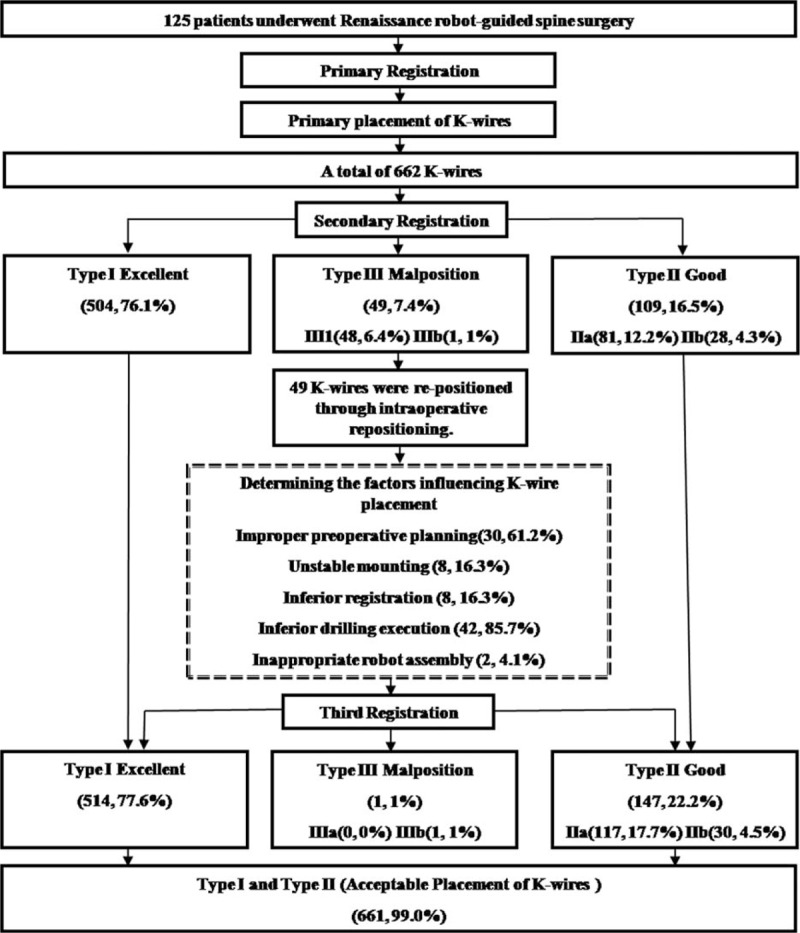
Procedures used for K-wire registration and repositioning.

The 125 Renaissance robot-guided spinal surgery procedures performed at Kaohsiung Medical University Hospital during the study period included 662 transpedicle screw implants and 49 Kirschner wire (K-wire) reimplants performed through intraoperative repositioning (Fig. 1). A K-wire intraoperative repositioning was defined as a deviation >3 mm according to the criterion for type III malposition in the Kuo et al (2016) classification scheme^[12]^ and incidental discovery of a slipped K-wire during surgery.

Data collection included all clinical presentations, particularly perioperative and postoperative status. Collection of postoperative follow-up data included transpedicle screw placement accuracy and functional outcomes.

### 2.4 Perioperative status

Surgical conditions included intraoperative blood loss, operating time, and intraoperative X-ray exposure time. Photographs taken during surgery were collected and analyzed. The mean operating time and mean intraoperative X-ray exposure time were 40.8 ± 18.8 and 7.2 ± 1.4 min, respectively (Table 1).

**Table 1:**
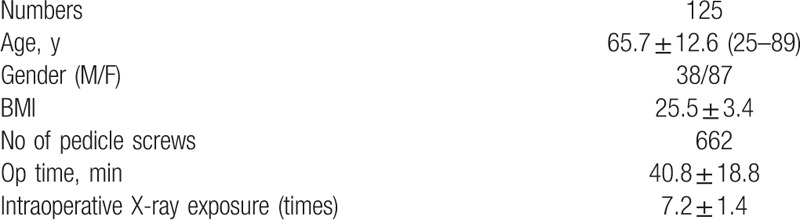
Clinical characteristics of 175 patients who underwent surgery through robotic-guided pedicle screw placement.

### 2.5 Robotic surgical techniques

All surgical procedures were performed by the lead author in this study (Dr. Tsai). The Renaissance robot-guided system is described in detail in Lieberman et al,^[11]^ Togawa et al,^[11]^ Devito et al,^[7]^ and Pechlivanis et al.^[6]^

The Renaissance robot-guided system was used to perform transpedicle screw placements in 4 main steps. *Preoperative planning:* Computed tomography (CT) scanning of the spine was performed preoperatively to reconstruct the 3D plane of the spine and to select the best screw placement strategy. *Mounting:* The mounting system was attached to an appropriate bony structure of the spine to maintain stability during registration. The robotic arm of the guided system was also fixed to the bony structure to provide intraoperative guidance. *Registration:* The Renaissance robot-guided system automatically registered the intraoperative images with the preoperative CT images by comparing 2 radiographic images (anteroposterior and oblique views). *Drilling:* The robot was attached to a mounting frame and moved to the position selected in preoperative planning. After directing the guiding tube at the pedicle, the surgeon began drilling. The robot was then used to insert the K-wire.

### 2.6 Classification of K-wire placement accuracy

The lead author classified the accuracy of all K-wire insertions relative to the transpedicle screw trajectory selected in preoperative planning as Types I–III in accordance with the Kuo et al^[12]^ classification scheme.

### 2.7 Causes of inaccurate K-wire placement

In patients who required pedicle screw repositioning after the registration step, the causes of inaccurate K-wire placement were determined according to the consensus of 3 experienced spinal surgeons. Causes of inaccurate K-wire placement were classified into 5 categories of errors: preoperative planning, mounting, registration, drilling, and robot assembly. Preoperative planning error was defined as an entry point at the slope of the bony structure, an insufficiently wide entry angle, or a preoperative in–out–in trajectory. Mounting error was defined as excessive movement of the mount relative to the vertebrae. Registration error was defined as a registration error exceeding 1 mm. Drilling error was defined as relative movement of vertebrae under excessive drilling pressure due to loss of cannula traction in soft tissue. [Robot assembly error was defined as drilling not performed according to standard procedures. Confusing.]

### 2.8 Postoperative status

#### 2.8.1 Postoperative accuracy of pedicle screw placement

Pedicle screw placement accuracy was classified into 4 categories according to postoperative biplanar fluoroscopy results.^[13,14]^

#### 2.8.2 Functional outcomes

Preoperative and postoperative Oswestry Disability Index (ODI) and visual analog scale (VAS) were used to evaluate functional outcomes.

##### 2.8.2.1 Statistical analyses

Paired *t* tests were used to compare function before and after surgery performed with the bone-mounted miniature robot system. McNemar test was used to compare accuracy before and after repositioning and before and after secondary registration. The analyses were performed with the SPSS 19.0 software package (SPSS Version 19.0 for Windows, IBM, Armonk, NY). A *P* value less than 0.05 was considered statistically significant.

## 3 Results

### 3.1 Intraoperative accuracy of K-wire placement

The actual trajectories of K-wires inserted through re-registration were compared with the planned trajectories (Table 2). The respective placement accuracies before and after repositioning were 76.1% and 77.6% in type I; 12.2% and 17.7% in type IIa; 4.3% and 4.5% in type IIb; 6.4% and 0% in type IIIa; and 1% and 1% in type IIIb.

**Table 2:**
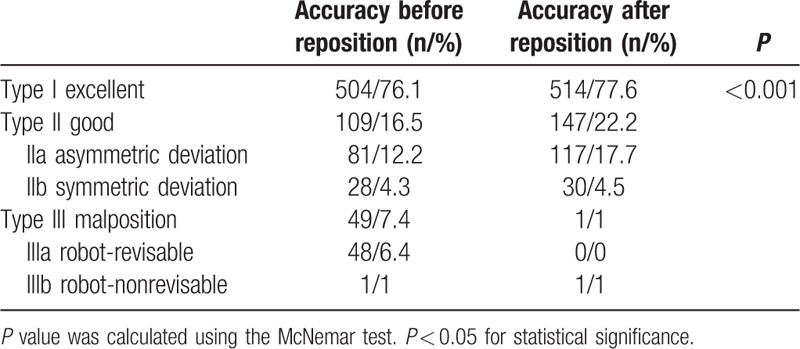
Classification of the accuracy of K-wire placement for 662 screws.

### 3.2 Factors in K-wire placement accuracy

**Figure 2:**
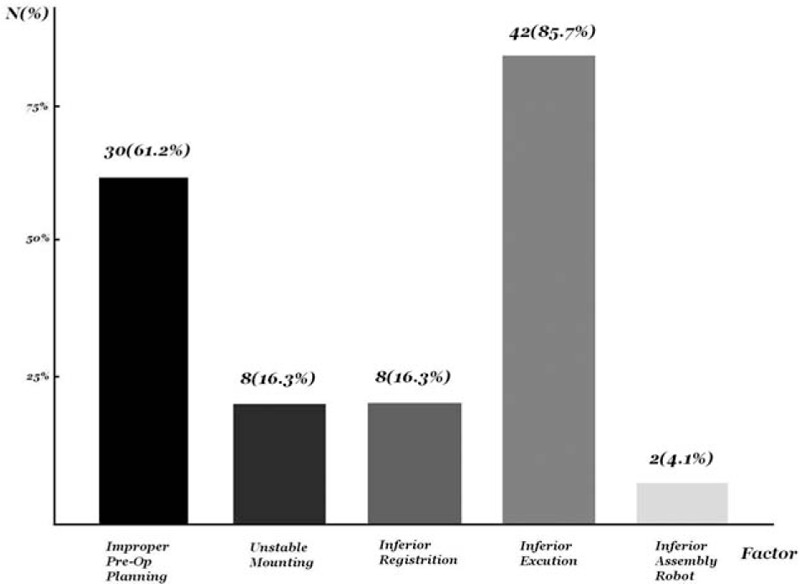
Comparison of factors that affect pedicle screw placement accuracy.

The accuracy of K-wire placement was decreased by errors in preoperative planning, mounting, registration, drilling, and robot assembly. The percentages of screws requiring repositioning were, from highest to lowest, 85.7% for drilling error, 57% for preoperative planning error, 17% for mounting error, 17% for registration error, and 2% for robot assembly error. That is, the most common cause of inaccurate K-wire placement was drilling error; drilling errors were attributable to robot assembly errors (Table 3, Fig. 2).

**Table 3:**
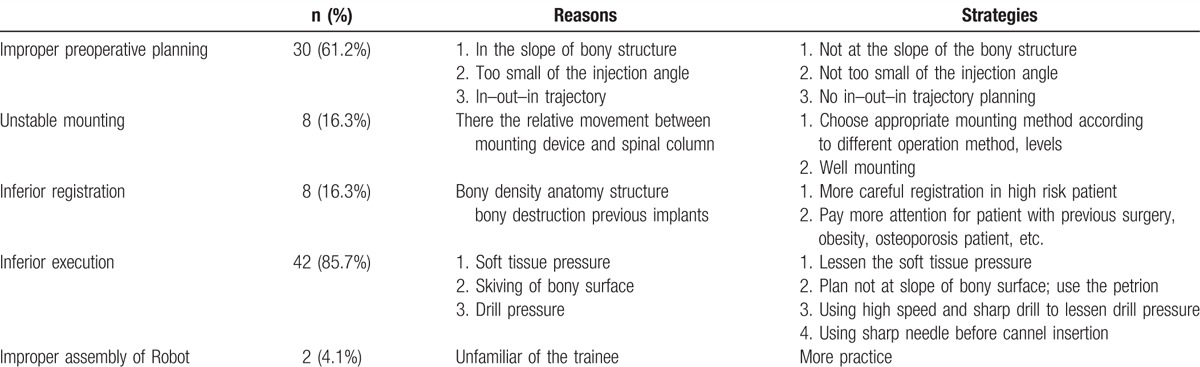
Factors influencing the placement of 49 repositioned K-wires.

### 3.3 Postoperative radiographic accuracy of pedicle screw placement

The accuracies of transpedicle screw insertions were as follows: 98.5% in Grade I; 1% in Grade II; 0.5% in Grade III; and 0% in Grade IV.

### 3.4 Functional outcomes

The preoperative and postoperative ODIs were 27.9 ± 6.1 and 13.1 ± 5.2, respectively, and the corresponding VAS scores were 7.2 ± 1.4 and 2.2 ± 1.2, respectively (Table 4). That is, comparison of preoperative and postoperative ODIs and VAS scores revealed significantly improved functional outcomes.

**Table 4:**
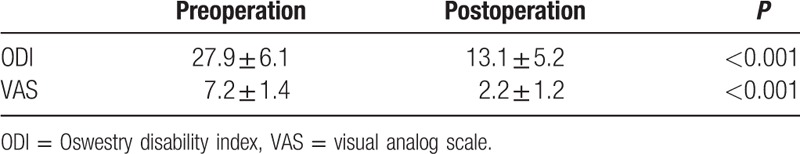
The functional outcomes of 125 patients with Robotic-guided pedicle screw placement.

## 4 Discussion

According to the literature, robot-assisted pedicle screw placement consistently obtains satisfactory accuracy and functional outcomes.^[9,15–17]^ In the present study, robot-assisted procedures obtained acceptable accuracy in 98.8% of K-wire placements and significantly improved functional outcomes after surgery. Therefore, the Renaissance robotic system not only improves precision and accuracy when used as an assistive tool for pedicle screw implantation, it also improves functional outcomes. However, the accuracy of pedicle screw placements performed with the Renaissance robot-guided system is still dependent on several factors. This article describes the 5 main factors and their respective effects on pedicle screw placement accuracy: preoperative planning, mounting, registration, drilling, and robot assembly (Table 3, Fig. 2).

### 4.1 Preoperative planning error

**Figure 3:**
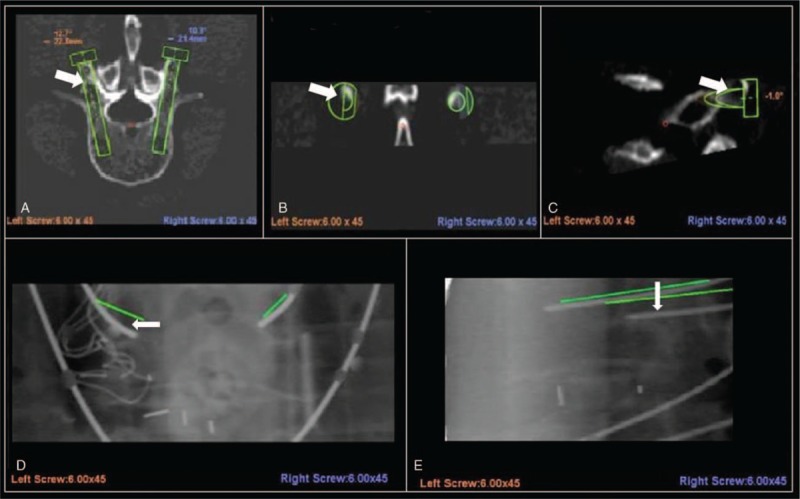
Preoperative planning error. Arrows in this figure parts (A–C) indicate the entry point selected in preoperative planning. The bony slope visible in the lateral inferior direction may be related to lateral inferior deviation. After the registration step, the left L5 K-wire lateral inferior deviation was >3 mm (type III, malposition).

Preoperative planning error includes selection of an inappropriate site for transpedicle screw placement. Deviations from the planned trajectory can occur when the facet joint surface is not sufficiently smooth and when the angle between the planned trajectory and the vertebrae is not sufficiently wide. Sliding can also occur if an improper in–out–in trajectory is selected during preoperative planning^[9]^. Specifically, an in–out–in trajectory can cause sliding in cases involving an extreme lateral facet joint. Hence, 57% (20/35) of the repositioned screws were associated with preoperative planning error (Fig. 3). Meticulous preoperative planning is thus crucial to ensure that the planned trajectory is not along the slope of the bony structure and that the projection angle is sufficiently wide. An in–out–in trajectory should also be avoided unless the pedicle is extremely small.

### 4.2 Mounting error

Deviation of screws from the planned trajectory can also occur when the mounting is not sufficiently stable. Roser et al^[17]^ hypothesized that unstable mounting can reduce screw placement accuracy. Hu et al^[14]^ and Lieberman et al^[8]^ further reported that insufficient fixation may cause relative movement between the robot and the patient. Schizas et al^[10]^ proposed that excessive drilling pressure can cause movement in the mount and pedicle. Minimizing the relative movement between the mounting device and the spinal column increases accuracy. In our series, the percentage of repositioned screws associated with unstable mounting was 17% (6/35). Depending on the operating method and various mounting methods can be employed. A Hover-T or multiuse clamp can be used initially for a long segment, and a bed-mount can be used for a short level. A clamp can then be used for a conventional median incision. To minimize movement of the mounting device relative to the spinal column, our strategy was to select the best mounting method for the individual patient and then enhance fixation during surgery.

### 4.3 Registration error

Potential causes of registration error include osteoporosis, various properties of the vertebral column, reoperation using implants, and destruction of bone in previous surgery. Pechlivanis et al^[6]^ reported that transpedicle screws cannot be inserted accurately if registration is inaccurate. Roser et al^[17]^ further reported that registration errors can result from inferior bone quality and from artifacts such as pacemakers or sternal wiring. In our series, the percentage of repositioned screws associated with registration errors was 17% (6/35). Hu et al^[14]^ further reported that registration errors can result from severe deformity, high body mass index, extremely low bone quality, and loose (previously implanted) hardware. Therefore, registration must be performed with extreme care in patients who have received prior surgery and in patients with obesity or osteoporosis.

### 4.4 Drilling error

**Figure 4:**
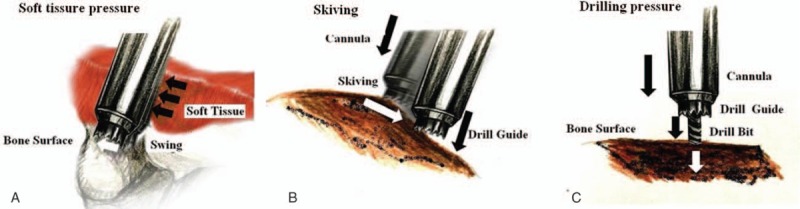
Accuracy of transpedicle screw placements can be affected by soft tissue pressure, bony surface skiving, and drilling pressure during insertion.

Three factors that affect transpedicle screw placement accuracy are excess *soft tissue pressure, bony surface skiving, and incorrect drilling pressure* (Fig. 4). The percentage of repositioned screws associated with drilling error in this study was 85.7% (30/35).

### 4.5 Excess soft tissue pressure

Soft tissue pressure can cause cannula traction injury associated with hazardous medial deviation (Fig. 4A). Our clinical experience shows that soft tissue pressure is minimal when pedicle screw placements are performed percutaneously and maximal when pedicle screw placements are performed by conventional midline approach. Our literature review further showed that the accuracy of the Renaissance robot-guided system for transpedicle screw placement is lower than that of conventional methods.^[9]^ The reduced accuracy results from soft tissue pressure causing traction of the cannula, which creates an inward force associated with potentially dangerous medial deviation. Devito et al^[7]^ proposed that deviation can be minimized by using a low soft tissue pressure; therefore, the access point must be carefully selected to minimize soft tissue pressure and the possibility of deviation.

### 4.6 Bony surface skiving

**Figure 5:**
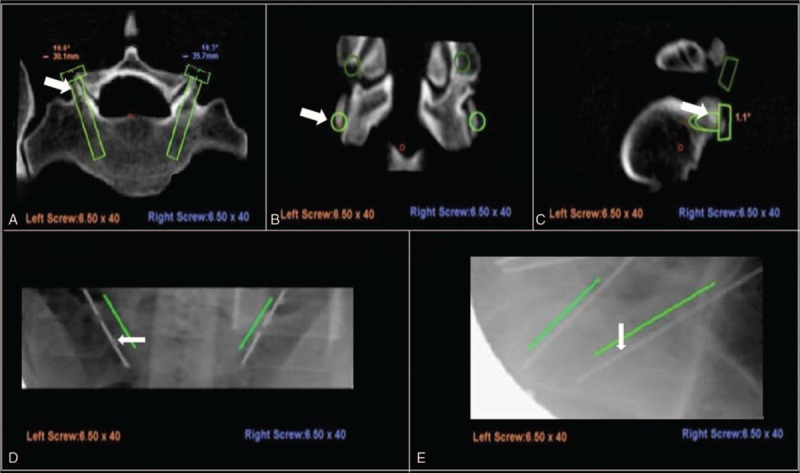
The entry point selected in preoperative planning was the slope of the bony surface (A–C). After reregistration, the right K-wire is type I (excellent), and the left K-wire is type IIIa (malposition, reversible) (D and E).

*Skiving* can result from either an irregular bony surface or a narrow angle between the trajectory and the vertical line (Fig. 4B). Hu et al^[14]^ reported that skiving can cause displacement of transpedicle screws. Ringel et al^[9]^ also reported that lateral skidding of the cannula can cause a steep slope in the facet joint (Fig. 5).

**Figure 6:**
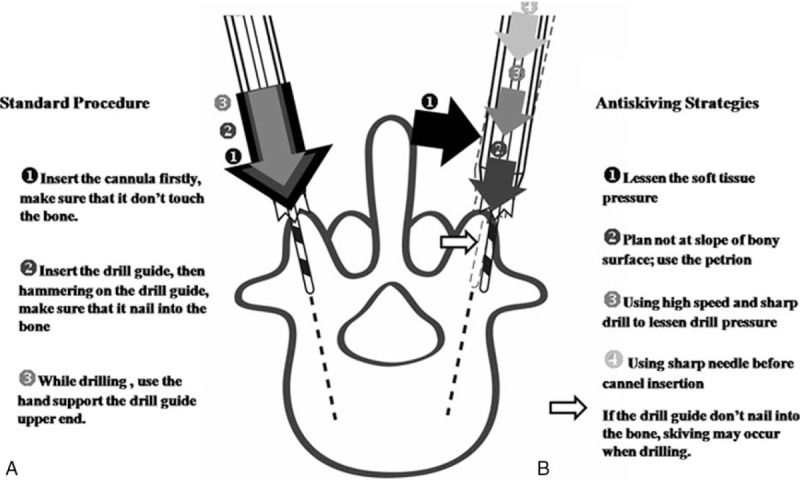
Demonstration of drilling procedure. Several strategies were used to prevent skiving. Part (A) shows the standard drilling procedure, in which a crown is inserted into the sheath before drilling. Part (B) depicts the antiskiving strategies.

Skiving can be minimized by choosing an entry point other than the slope of the bony surface, by using a high-speed drill with a sharp bit for drilling, and by using an antiskiving pin (Fig. 6).

### 4.7 Incorrect drilling pressure

Insufficient drilling pressure can result from using a blunt drill bit, an excessively low drilling speed, or an inappropriate mounting. Pressure may also be insufficient when the drilling site is a hard bony structure (Fig. 4C). In contrast, excessive drilling pressure can displace vertebrae. Schizas et al^[10]^ reported that excessive drilling pressure can cause relative sliding of the bony structure. As a result, the mounting device may induce a deviation at the entry point. Drilling pressure can then push the vertebrae away from the mounting system (i.e., the drawbridge effect). The drawbridge effect can be avoided by minimizing drilling pressure through the use of a high-speed drill with a sharp bit and the appropriate mounting system.

### 4.8 Robot assembly error

Human error can lead to unfavorable results. The proportion of intraoperatively repositioned K-wires was 2/35 (5.7%). The improper positioning was attributable to deviations caused by inappropriate robot assembly procedures. Two patients had no problems during the operation; however, a records review showed that the K-wire positions were approximately 1 level higher than the target position. This error resulted from use of the wrong station during robot assembly procedures. Errors in the assembly procedures for the robot-guided system can cause catastrophic neurological damage and massive bleeding.

## 5 Limitations

The gold standard for evaluating pedicle screw placement accuracy is CT. However, this study evaluated placement accuracy by using an intraoperative robotic classification system. However, postoperative CT^[12]^ also proved to be a feasible method for evaluating K-wire placement accuracy and for using an intraoperative robot grading system to predict postoperative accuracy of pedicle screw placements.^[18]^

Factors that affected the accuracy of transpedicle screw placement were categorized as either robotic or nonrobotic factors. Factors that negatively affected robot-assisted pedicle screw placement were identified according to the consensus of 3 experienced spinal surgeons and divided into 5 categories of errors: preoperative planning, mounting, registration, drilling, and robot assembly. However, this study did not consider nonrobotic factors such as osteoporosis, obesity, and prior spinal surgery. Thus, further studies are needed to evaluate the roles of these factors.

Another potential limitation is that, to exclude intraobserver bias, the accuracy of pedicle screw placement using a bone-mounted miniature robot system was only analyzed in robotic spinal surgery procedures performed by a single surgeon. However, differences in surgical skills, experience, and technologies may increase or decrease certain errors. That is specific error types may be associated with specific levels of skill and experience. The factors affecting pedicle screw placement accuracy were only discussed in relation to a single surgeon in this study, which raises the issue of interobserver bias. Further studies are needed to compare factors that affect the accuracy of pedicle screw placements performed by different surgeons (intraobserver bias).

## 6 Conclusions

In summary, this study of factors that affect pedicle screw placement accuracy showed that inaccurate pedicle screw placement can result from errors in preoperative planning, mounting, registration, execution, and robot assembly. Pedicle screw placement using a bone-mounted miniature robot system must be performed with extreme care to avoid these errors.
